# Chicken liver is a potential reservoir of bacteriophages and phage‐derived particles containing antibiotic resistance genes

**DOI:** 10.1111/1751-7915.14056

**Published:** 2022-04-29

**Authors:** Pedro Blanco‐Picazo, Clara Gómez‐Gómez, Sergi Aguiló‐Castillo, Dietmar Fernández‐Orth, Marta Cerdà‐Cuéllar, Maite Muniesa, Lorena Rodríguez‐Rubio

**Affiliations:** ^1^ Department de Genètica, Microbiologia i Estadística Universitat de Barcelona Diagonal 643, Planta 0 Barcelona 08028 Spain; ^2^ Spanish National Bioinformatics Institute (INB)/ELIXIR‐ES Barcelona Supercomputing Center Barcelona Spain; ^3^ Department of Bioinformatics and Molecular Biology Cerba Internacional Pl. Ramon Llull 7‐10, Sabadell Barcelona 08203 Spain; ^4^ IRTA Centre de Recerca en Sanitat Animal (CReSA, IRTA‐UAB) Campus de la Universitat Autònoma de Barcelona Bellaterra, Barcelona 08193 Spain

## Abstract

Poultry meat production is one of the most important agri‐food industries in the world. The selective pressure exerted by widespread prophylactic or therapeutic use of antibiotics in intensive chicken farming favours the development of drug resistance in bacterial populations. Chicken liver, closely connected with the intestinal tract, has been directly involved in food‐borne infections and found to be contaminated with pathogenic bacteria, including *Campylobacter* and *Salmonella*. In this study, 74 chicken livers, divided into sterile and non‐sterile groups, were analysed, not only for microbial indicators but also for the presence of phages and phage particles containing antibiotic resistance genes (ARGs). Both bacteria and phages were detected in liver tissues, including those dissected under sterile conditions. The phages were able to infect *Escherichia coli* and showed a Siphovirus morphology. The chicken livers contained from 10^3^ to 10^6^ phage particles per g, which carried a range of ARGs (*bla*_TEM_, *bla*_CTx‐M‐1_, *sul1*, *qnrA*, *armA* and *tetW*) detected by qPCR. The presence of phages in chicken liver, mostly infecting *E. coli*, was confirmed by metagenomic analysis, although this technique was not sufficiently sensitive to identify ARGs. In addition, ARG‐carrying phages were detected in chicken faeces by qPCR in a previous study of the group. Comparison of the viromes of faeces and liver showed a strong coincidence of species, which suggests that the phages found in the liver originate in faeces. These findings suggests that phages, like bacteria, can translocate from the gut to the liver, which may therefore constitute a potential reservoir of antibiotic resistance genes.

## Graphical abstract

Phage particles carrying antibiotic resistant genes have been found in chicken livers. Metagenomic analysis of chicken liver viromes (PL) and chicken faeces viromes (HP) suggests that these phage particles could translocate from chicken gut to the liver.

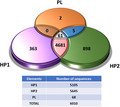

## Introduction

The European Union (EU) is one of the world’s largest producers of poultry meat and a net exporter of poultry products, with an annual production of around 13.4 million tonnes (Poultry | European Commission). Poultry is the only meat category whose production has expanded during the Covid‐19 pandemic and the only one expected to grow between 2020 and 2030 in the EU (European Commission). The consumption of poultry meat is also projected to increase on a global scale (OECD‐FAO AGRICULTURAL OUTLOOK 2018–2027), as it is regarded as a cheap, healthy and sustainable product by consumers.

Intensive poultry production, one of the fastest growing industries in the world, strongly depends on antimicrobials to prevent or treat diseases. In several countries, including Spain, the poultry production sectors are committed to a responsible use of antibiotics and are gradually reducing their application (Programa REDUCE Pollos Broiler | PRAN). Nevertheless, in other parts of the world, the usage of antibiotics as prophylactics, or even as growth promoters, a forbidden practice in the EU and USA, is difficult to eradicate due to the fear of infections spreading through the poultry farms. The consequence of such an intensive use of antibiotics is a very high selective pressure on bacterial populations (Xiong *et al*., 2018), which promotes mutations, the emergence of antibiotic resistances and the dissemination of antibiotic resistance genes (ARGs) by mobile genetic elements (World Health Organization, 2018). Many pathogenic or commensal bacteria isolated from poultry encode antimicrobial resistances (Nhung *et al*., 2017), which can be transmitted to humans by direct or indirect contact (e.g., through food, water and the environment). Multi‐drug resistant bacteria arising from animal production practices have been found in everyday consumer products (Antimicrobial Resistance in the Environment Summary Report of an FAO Meeting of Experts FAO Antimicrobial Resistance Working Group, 2018).

Among the diverse elements that drive the mobilization of ARGs, the most extensively studied are plasmids and transposons (Brown‐Jaque *et al*., 2015). Recently, phages and phage‐derived particles have been recognized as underestimated mobile genetic elements responsible for ARG transfer and as ARG reservoirs in the environment (Colomer‐Lluch *et al*., 2011a; Ross and Topp, 2015; Subirats *et al*., 2016; Blanco‐Picazo *et al*., 2020). First discovered in the second decade of the 20th century (D’Herelle, 1917), phages have been receiving renewed attention for their role as regulators of bacterial populations in natural ecosystems (Fuhrman, 1999). Many metagenomic analyses of human and animal microbiomes have described the extremely high abundance of phages, generally greater than that of eukaryotic viruses (Breitbart *et al*., 2003; Colomer‐Lluch *et al*., 2011b; Minot *et al*., 2011; Virgin, 2014). Phages have been reported in the lung, vaginal, skin, oral or intestinal microbiota (Oh *et al*., 2014) and more recently, infectious phages have been found in clinical samples, such as ascitic fluids and urine (Brown‐Jaque *et al*., 2016). It has been shown that phages can translocate from the intestinal tract to the peritoneal cavity, bloodstream and other organs (Górski *et al*., 2006; Dąbrowska, 2019; Podlacha *et al*., 2021). In animals, phages infecting *Bacteroides* have been found in serum (Keller and Traub, 1974) and phages infecting *E. coli* O157 show good persistence in the circulatory system of mouse (Capparelli *et al*., 2006). Also, phages translocate from blood to mouse foetal tissues in pregnant mice (Srivastava *et al*., 2004) and from intestinal tract to blood in Goldfish (Kawato and Nakai, 2012).

Considering that phages are found in different systems and organs of the human and animal bodies (Dąbrowska, 2019; Kaźmierczak *et al*., 2021; Podlacha *et al*., 2021), and that those carrying ARGs have been detected in different human systems (Brown‐Jaque *et al*., 2016; Blanco‐Picazo *et al*., 2020), ARG‐carrying phages are also expected to occur in animal organs. In chickens, the largest source of meat intended for human consumption, the liver is of particular concern as it is closely connected with the gastrointestinal tract and has been identified as a vehicle for food‐borne infection (Lanier *et al*., 2018). Pathogens such as *Campylobacter* or *Salmonella* are thought to spread from the gastrointestinal tract to the liver through the biliary, lymphatic, or vascular systems (Lanier *et al*., 2018). The ready detection of bacterial pathogens in internal tissues of chicken liver (Firlieyanti *et al*., 2016; Lanier *et al*., 2018), and the numerous studies of the circulation of phages in different organs (Kaźmierczak *et al*., 2021), including liver (Rusckowski *et al*., 2008; Dąbrowska, 2019; Podlacha *et al*., 2021) suggests that phages containing ARGs can also be present in the liver. To date, ARG‐carrying phages in chicken have only been confirmed in meat samples and faeces (Gómez‐Gómez *et al*., 2019).

In this study, internal tissues of chicken liver samples were analysed for the presence of phages using a metagenomics approach and the liver virome was compared with that of faecal samples. Infectious phages were evaluated in the same liver samples. Finally, ARG‐carrying phages in total liver and internal tissues were analysed by quantitative real‐time PCR (qPCR).

## Results and discussion

A high percentage of non‐sterile liver samples contained moderate amounts of culturable microbial indicators (Table 1). To confirm that these indicators originated from the liver tissues and were not the result of contamination from the peritoneal cavity, a second set of livers were analysed in which the inner tissues were dissected under more strict sterile conditions, thus avoiding the potentially contaminated external parts. Although fewer sterile livers contained culturable microorganisms, when present, the average values were not remarkably lower (Table 1), suggesting that the detected bacteria and phages, at least partially, did not originate from external contamination. Somatic coliphages confirmed the presence of phages infectious for the *E. coli* WG5 strain.

**Table 1:** Bacterial and viral indicators in the chicken liver samples.

Sample	Value	Somatic coliphages (PFU g^−1^)	Total aerobic bacteria (CFU g^−1^)	Ampicillin‐resistant total aerobic bacteria (CFU g^−1^)
Non‐sterile liver	*n*	47	47	47
	% Positive	66.0	100.0	95.0
	Average	2.6 × 10^2^	1.5 × 10^4^	2.1 × 10^3^
	Max–min	10^3^–5	2.2 × 10^5^–4.2 × 10^3^	2.0 × 10^3^–5.0 × 10^2^
Sterile liver	*N*	27	27	27
	% Positive	60.6	96.4	67.9
	Average	2.2 × 10^2^	1.2 × 10^4^	1.5 × 10^3^
	Max–min	10^3^–5	1.6 × 10^5^–5.0 × 10^2^	2.7 × 10^3^–5.0 × 10^1^

### Observation of phage particles in sterile liver samples

**Fig. 1:**
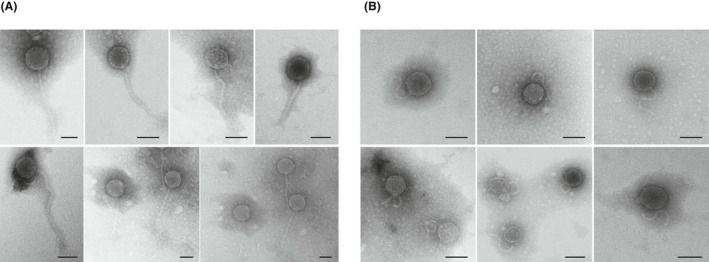
Transmission electron micrographs of phages purified from a pool of 10 chicken liver samples from group 2 (sterile inner tissue). Two morphologies were observed: phages with icosahedric capsids and a long non‐contractile tail (A) or a short and curly non‐contractile tail (B). Bar 50 nm.

To confirm these results, total phage particles in sterile livers were monitored by TEM. As titres of at least 10^7–8^ PFU are necessary for phage detection by electron microscopy (Brown‐Jaque *et al*., 2016), a pool of phage suspensions from 10 livers were 100× concentrated as described in the methods section and used for TEM observation. Phage particles with icosahedral heads of 57–62 nm and non‐contractile tails were observed (Fig. 1). In the same pool, the visualized phages had long straight tails (Fig. 1A) as well as curly tails, suggesting that the concentrated samples contained at least two different types of phages (Fig. 1B). It should be noted that the concentration step and the requirement for high titres of phages for TEM observation (Blanco‐Picazo *et al*., 2020) may have reduced the diversity of observable phages to those reaching densities above 10^7–8^ PFU.

### Presence of ARGs in the phage DNA fraction of chicken liver

**Fig. 2:**
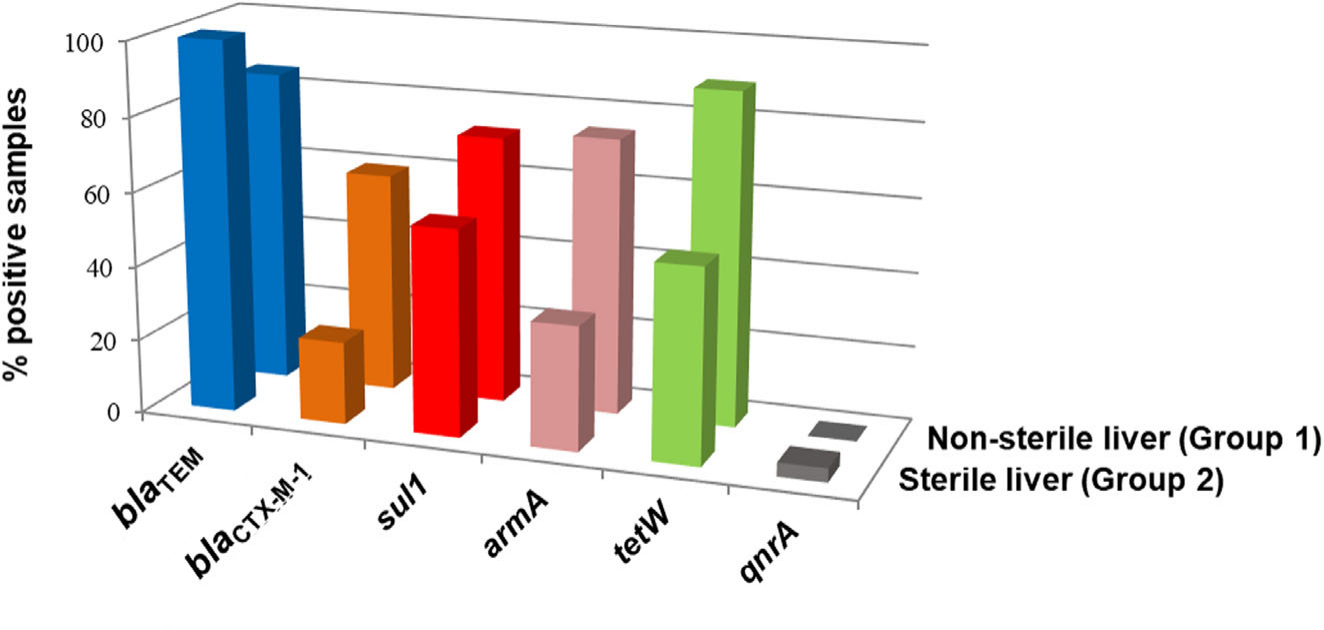
Antibiotic resistance genes (ARGs) in the phage DNA fraction of chicken liver samples. Percentage of positive samples for each ARG (*bla*_TEM_, *bla*_CTX‐M‐1_, *sul1*, *armA* and *tetW*) in each matrix. Two types of samples were analysed; group 1: the whole liver (non‐sterile) and group 2: inner tissues of the liver (sterile), recovered by dissection with a sterile scalpel.

**Fig. 3:**
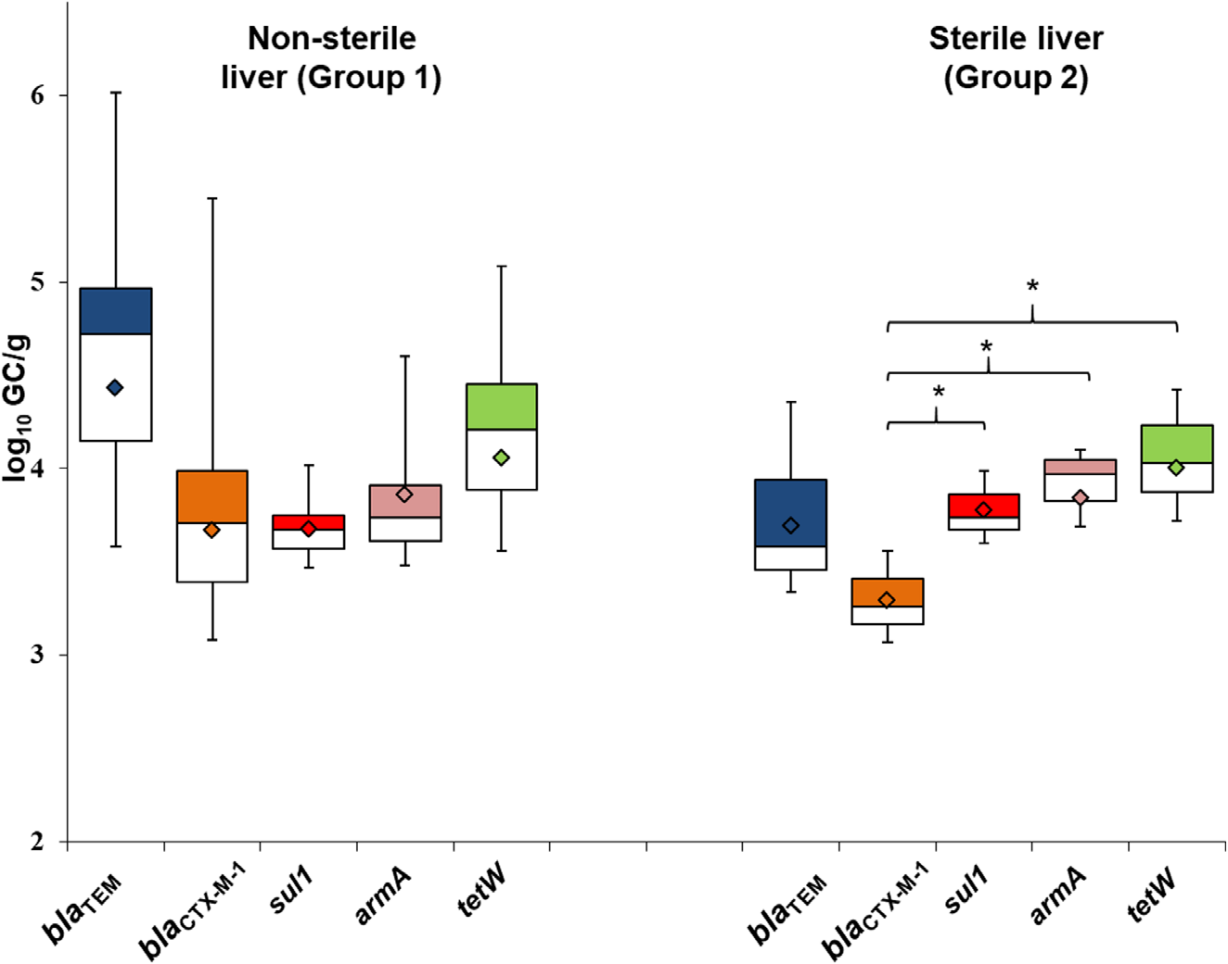
Abundance of each antibiotic resistance gene (ARG) in phage DNA isolated from chicken liver samples of group 1: non‐sterile (47 samples) and group 2: sterile (27 samples). Box plot of the average values (log_10_ GC g^−1^ sample) of all ARGs in the positive samples. The cross‐pieces of each box represent (from top to bottom) the maximum, upper‐quartile, median, lower quartile, and minimum values. The diamond (black) shows the mean value. The upper boxes (white) in the box plot include samples showing values within the 75th percentile and lower box samples (grey) show values within the 25th percentile.

Antibiotic resistance genes were detected in the phage DNA fraction of a large percentage of liver samples, with variable results for the five targeted ARGs (Fig. 2), irrespective of whether the livers were sterile or not. Except for *bla*_TEM_, ARG prevalence was lower in the viral fraction of sterile (Fig. 2) versus non‐sterile liver. *qnrA* was only detected in sterile liver, and only in one sample (*qnrA* is not included in Fig. 3). This single positive does not allow conclusions to be drawn about the prevalence of *qnrA* in the two groups.

Antibiotic resistance gene abundance (Fig. 3) did not differ significantly (*P* > 0.05) between sterile and non‐sterile livers. In non‐sterile liver, there were no significant differences (*P* > 0.05) between the abundances of the different ARGs. In the sterile liver, the abundance of *bla*_CTX‐M‐1_ showed significant (*P* < 0.05) differences when compared with *armA*, *sul1*, or *tetW*, respectively. As *qnrA* was only detected in one sterile liver sample, it was not included in the chart. 3.07 log_10_ of *qnrA* gene copies were detected per g of liver.

### Liver and faecal viromes

**Fig. 4:**
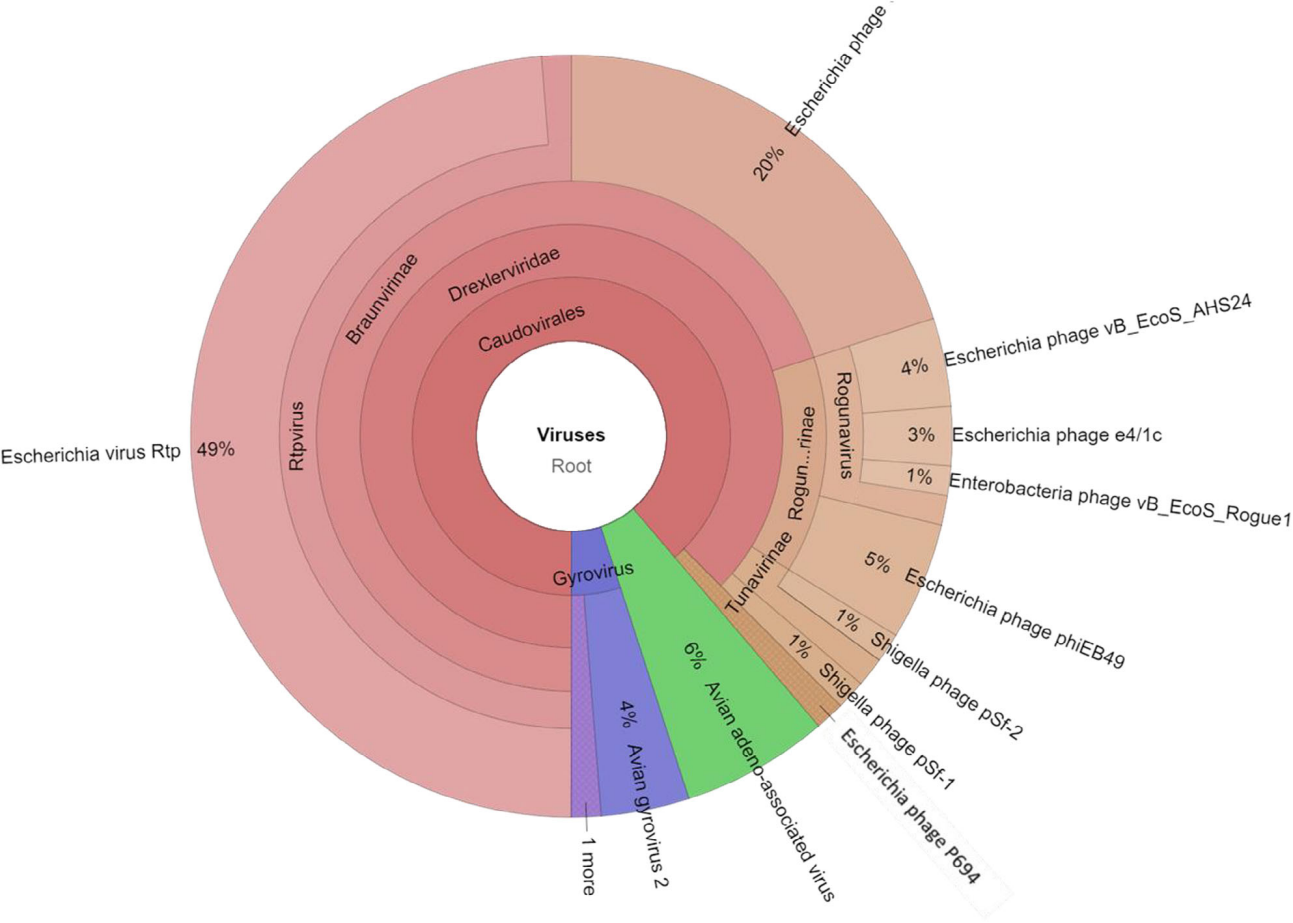
Pie graphs showing the diversity of the viral fraction of chicken liver simples according to Kraken classification of metagenomics data. The graphs compare the distribution and relative abundance of viruses identified in the viromes with regard to the total viral sequences identified. Interactive pie graphs are available upon request.

The viromes of a pool of sterile liver samples were analysed to gain more insight into the phage fraction. The purification of encapsidated DNA to achieve the quality required for metagenomics resulted in the generation of a relatively low amount of contigs; 62% of the sequences were unclassified when compared against general databases, and did not correspond to eukaryotic, archaeal or bacterial DNA, while the rest of contigs were identified as bacterial (28%) and viral (11%) (Table 2). The great majority of viral sequences (89%) corresponded to bacteriophages of the Caudovirales order, particularly of the *Drexlerviridae* family (Fig. 4), which comprises phages with an icosahedral head and long non‐contractile tail, compatible with the TEM observations (Fig. 1). Most phages in the liver virome showed homology with *E. coli* or *Shigella* phages, although the high number of sequences of Enterobacteria phages, particularly those of *E. coli*, in the databases may have influenced this result. Considering the widespread genome mosaicism reported in phages (Hatfull, 2008), those infecting Enterobacteria should share several DNA fragments. The non‐phage viral sequences corresponded to gyroviruses (5%) and avian adeno‐associated viruses (6%) (Fig. 4).

**Table 2:** Metagenomic parameters in chicken liver and chicken faecal samples.

Origin	#contigs/Total length (bp)	Size of the longer contigs (bp)	#contigs Unclassified (%)	#contigs Archaea (%)	#contigs Bacteria (%)	#contigs Viruses (%)
Chicken liver (PL)	11 539 / 164 020	1646	7121 (61.71)	5.8 (0.05)	3196 (27.70)	1216 (10.54)
Chicken faeces (HP1)	643 753 / 295 827 925	110 470	535 005 (83.11)	329 (0.05)	105 241 (16.35)	2638 (0.41)
Chicken faeces (HP2)	1 163 538 / 558 526 824	237 483	954 610 (82.04)	611 (0.05)	204 160 (17.55)	3282 (0.28)

Although phage presence in the analysed samples was confirmed and ARGs were detected in the packaged DNA by qPCR, metagenomic sequencing of the same DNA was of insufficient depth to identify any ARGs. Less sensitive than qPCR, metagenomics cannot determine genes present in moderate concentrations, such as ARGs, particularly in complex samples (e.g., liver, food and faeces) (Fernández‐Orth *et al*., 2019; Blanco‐Picazo *et al*., 2020). On the other hand, not being limited to specific genes, metagenomics can identify a more extensive repertoire of ARGs. It should also be mentioned that the successful elimination of all traces of non‐packaged DNA, which required additional steps in the protocol, was achieved at the cost of reducing the recovery and quality of the extracted DNA.

The presence of phages (including those carrying ARGs) infectious for Enterobacteria, particularly *E. coli*, in the liver virome suggests a translocation from the intestinal virome (Górski *et al*., 2006; Brown‐Jaque *et al*., 2016; Chen *et al*., 2018). To gain further information, two chicken faecal viromes were analysed using the same protocol for phage purification and DNA extraction. The first finding was that the faecal virome contained a significantly higher amount of DNA, and consequently more sequences, compared with the liver virome (Table 2). However, a higher percentage of sequences remained unclassified, and a lower percentage were identified as viral (Table 2). Within the viral fraction, most bacteriophages were of the Caudovirales order (93% in HP1 and 88% in HP2) (Table S2).

A great diversity of ARGs was detected in the viromes of chicken faeces in accordance with the qPCR results obtained in the previous studies (Gómez‐Gómez *et al*., 2019) (Table 3) and in accordance with the high amount of ARGs in the faecal virome of humans (Fernández‐Orth *et al*., 2019) and viromes of faecally polluted environments (Colombo *et al*., 2016; Lekunberri *et al*., 2017; Chen *et al*., 2021; Wang *et al*., 2021). Only two ARGs were detected by both the metagenomic analysis (using either Prokka, CARD or Resfinder) and qPCR, *bla*_TEM_ and *tetW*, which were the most abundant (more than 6 and 8 log_10_ GC/g, respectively) (Table 3) according to the qPCR results. The other ARGs targeted by qPCR went undetected by the metagenomic approach, regardless of the bioinformatic platform used.

**Table 3:** ARGs in chicken faeces detected by metagenomics compared with the ARGs detected by qPCR.

	METAGENOMICS		qPCR	
	Gene	Resistance to	Average (log GC g^−1^)	Resistance to
HP1	*tet*M (1‐2)^a^, *tet*32, *tet*40, ***tet*W**	Tetracycline	***tet*W(8.55)**	Tetracycline
	***bla*_TEM_**	Betalactamics	***bla*_TEM_ (6.33)** *bla*_CTX‐M‐1_ (5.20)	Betalactamics
	*ant*(6)‐Ia, *aph*(2'')‐Ig, *aph*(3')‐III, *aac*(6')‐Im	Aminoglycoside	*arm*A (3.54)	Amynoglycoside
	*drrA* (2‐15)	Daunorubicin/doxorubicin	*qnr*A (3.63)	Quinolone
	*linA*	Lincosamide	*sul*1 (5.59)	Sulphonamide
	*mec*I (1‐3)	Methicillin		
	*mup*B	Mupirocin		
	*van*A (1‐2), *van*B (1‐3), *van*C (1‐2)	Vancomycin A, B, C‐type		
	*lnu*C	Lyncomycin		
	*cfr*C	Linezolid		
	*erm*B	Macrolide		
	*cat*	Chloramphenicol		
HP2	*tet*32(1‐2), *tet*M(1‐3), *tet*O, ***tet*W**	Tetracycline	***tet*W (8.00)**	Tetracycline
	***bla*_TEM_**	Betalactamics	***bla*_TEM_ (6.64)** *bla*_CTX‐M‐1_ (5.12)	Betalactamics
	*aph*(2'')‐Ig, *ant*(6)‐Ia, *aph*(3')‐III, *aac*(6')‐Im	Aminoglycoside	*arm*A (4.76)	Aminoglycoside
	*ble*	Bleomycin	*sul*1 (5.85)	Sulfonamide
	*drr*A(1‐34)	Daunorubicin/doxorubicin	*qnr*A (4.10)	Quinolone
	*cat*	Chloramphenicol		
	*cfr*C	Linezolid		
	*erm*B, *mef*A, *erm*G	Erythromycin		
	*lnu*C	Lyncomycin		
	*fsr*	Fosmidomycin		
	*lin*A (1‐2)	Lincosamide		
	*mec*I(1‐16)	Methicillin		
	*mup*A, *mup*B (1‐2)	Isoleucine‐‐tRNA ligase. Mupirocin		
	*tmr*B (1‐2)	Tunicamycin		
	*van*A (1‐13), *van*B (1‐6),*van*C (1‐8), *van*W (1‐8)	Vancomycin A, B and C‐type		

In bold, ARGs detected in the virome by metagenomics (either by Prokka or Resfinder) as well as by qPCR

^a^ In brackets are indicated the range of gene subtypes.

Several ARGs other than those targeted by qPCR were determined in the faecal viromes, conferring resistances to a range of antibiotics (e.g., chloramphenicol, macrolides, lincosamide and vancomycin) (Table 3). Resfinder, a more restrictive database, gave fewer results than Prokka or CARD, which also identified transporters. Since ABC transporters confer unspecific resistance to drugs, and cannot be classified as ARGs, they have not been included in these results despite they can be horizontally mobilized, either by phage particles or by plasmids (Kadlec and Schwarz, 2009).

**Fig. 5:**
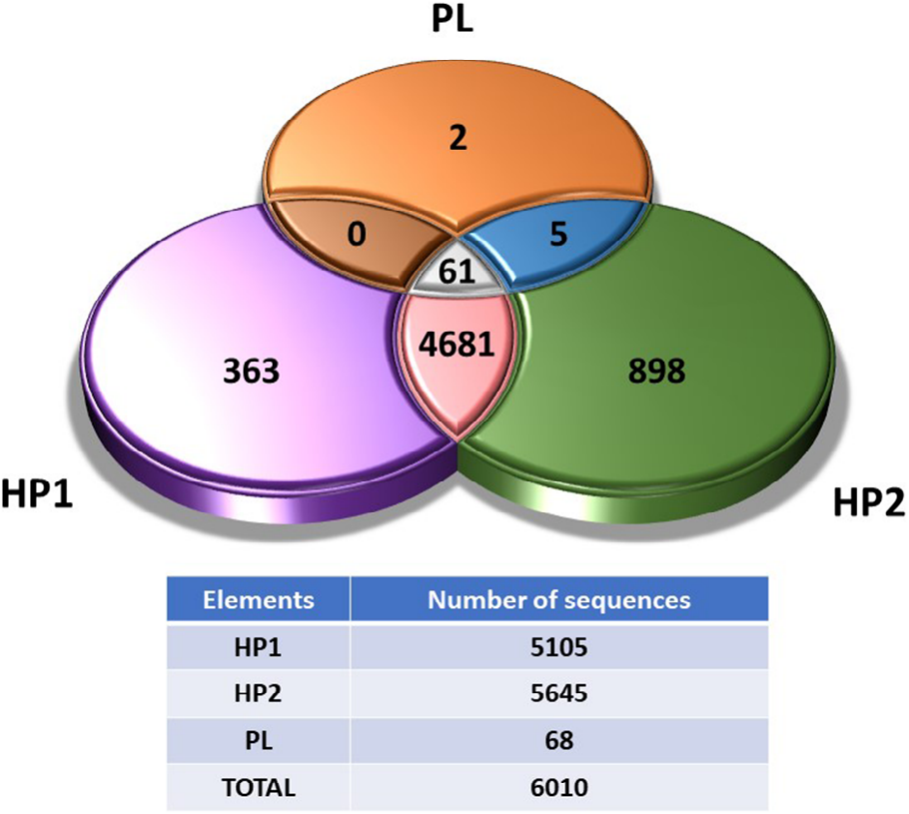
Venn diagram showing the number of unique and sharing sequences between the chicken liver virome (PL) and the chicken faecal viromes HP1 or HP2 respectively.

The viromes show a large set of bacterial sequences. Since the controls used have discarded that bacterial DNA contamination might have contributed significantly to these results, our assumption supported by previous studies (Fernández‐Orth *et al*., 2019; Fillol‐Salom *et al*., 2021; Humphrey *et al*., 2021) is that phages package bacterial DNA from their hosts more frequently than previously thought. This information, together with the sequences of viral species, can then be used to determine the route of transmission of these phages. Since we speculate that phages can come from faecal microbiomes, we searched for coincidences between the chicken liver virome (PL) and two different faecal viromes (HP1 and HP2). When comparing bacterial and viral species detected in the liver virome with those detected in the faecal viromes, 61 of the 68 (89.7%) species found in the PL virome were also present in HP1 (Fig. 5, Table S3). The seven absent organisms were *Cloacibacterium normanense*, Rogunavirus, Rtpvirus, *Escherichia* phage vB_EcoS_AHS24, Enterobacteria phage vB_EcoS_Rogue1, avian gyrovirus 2 and chicken anaemia virus. Similar results were observed when comparing the PL virome with HP2 (Fig. 5, Table S3), with 97% of the species (66 of the 68) in chicken liver being found in the faecal virome, only avian gyrovirus 2 and chicken anaemia virus being absent.

The high proportion of species in chicken liver virome matching those found in the faecal viromes (Fig. 5) points towards an intestinal origin of the viruses found in the chicken liver. It should be noted that although livers and faecal samples come from the same farm and type of chicken, samples were taken at different times, making the coincidence even more relevant. Translocation from the intestinal tract seems more likely than phages reaching the liver through systemic colonization. High accumulation of phages in liver tissues have been previously demonstrated (Dąbrowska, 2019; Van Belleghem *et al*., 2019; Kaźmierczak *et al*., 2021; Podlacha *et al*., 2021). However, pharmacokinetics studies suggest that although phages accumulate very efficiently in the liver, they are also quickly inactivated because liver is the first responsible of bacteriophage clearance (Dąbrowska, 2019; Van Belleghem *et al*., 2019), particularly by the macrophages found in the Kupffer cells (Inchley, 1969). Therefore, doubts arise whether the liver could be a reservoir of phages able to transduce ARGs to active recipient cells, despite the high accumulation. Noteworthy is the fact that the removal of phages in liver has been evaluated using phage suspensions, usually of a single phage type administered in animal models and not using resident phages. Mutant phages able to circumvent the mononuclear phagocyte system have been reported (Merril *et al*., 1996); therefore, if phages resistant to macrophages can be selected under laboratory conditions, it is plausible that natural subpopulations of phages with natural resistance to phagosomal degradation can be selected as well. In addition, the efficiency of the circulating phages removal and the tolerance of the immune system depend on many variables, such as the type of phages, the size of the virions, the amount of phages and even the administration method, if any (Dąbrowska, 2019; Podlacha *et al*., 2021). All this together could explain the presence of infectious phages in the livers analysed in this study.

Assuming that a fraction of the phage population could be retained within the inner tissues of the liver for a long enough period of time to inject their DNA if a suitable host is found, what happens next will depend on whether the phages are complete (with the entire phage genome in the capsid) or transducing particles (phage capsids containing only bacterial DNA). Phages can propagate in the host strain and transduce some of the genes they encode (including ARGs), whereas transducing particles can inject their DNA in a recipient cell, which can acquire the genes through recombination. ARG transfer to a suitable host is possible in both scenarios. Administering a strong antibiotic treatment to an animal colonized by bacteria transduced by an ARG‐carrying phage would impose strong selective pressure in favour of the transduced strains.

Chicken liver is a reservoir of *Campylobacter* and *Salmonella* (Firlieyanti *et al*., 2016; Lanier *et al*., 2018; Laconi *et al*., 2021) and also of avian pathogenic *E. coli* (Abdelhamid *et al*., 2020). Moreover, *Campylobacter* gastrointestinal colonization in chickens seems to contribute to the translocation of *E. coli* from the gut to extra‐intestinal organs, leading to a high prevalence of *E. coli* in the spleen and liver (Awad *et al*., 2016). Like their bacterial hosts, phages might easily translocate outside the intestinal environment to other organs, which can serve as reservoirs of phage particles and their genetic content and constitute environments for the emergence of new resistant strains.

## Experimental procedures

This study did not include animal experimentation. Animal liver samples were collected at the slaughterhouse at the time of sacrifice following the guidelines of the Ethical Committee for Animal Experimentation (CEEA), the authorized body by the Catalan Government. The guidelines are defined in European Parlament 2010/63/EU; RD 53/2013; Directive 2010/63/UE; Decret 214/1997/GC, Ordre ECC/566/2015 and regulate the use of animals for experimentation and other scientific purposes. All experimental procedures were conducted in accordance with the biosafety regulations for microorganisms and approved by the Office of Safety, Health and the Environment (OSSMA) of the University of Barcelona.

### Samples

Sampling was performed in two slaughterhouses from northeastern Spain between February and September 2020. Healthy animals with no prophylactic antibiotic treatment were randomly selected. During the evisceration process, a first group of livers was collected from carcasses. Whole livers were removed aseptically using clean gloves and ethanol‐disinfected scissors and placed in new, clean plastic bags. The livers of three carcasses per flock were pooled and analysed as a single sample. These correspond to group 1 comprising 47 whole liver samples.

However, after the analysis of culturable microorganisms, the high levels of bacteria and phages detected raised doubts about possible contamination from the peritoneal cavity. Therefore, a second group of samples was collected for this study (group 2). Group 2 comprised 27 internal liver tissue samples, each sample is a pool of three internal liver tissues from the same flock. To sample internal tissues, livers were placed on a sterile Petri dish and external parts of the liver were sanitized by searing the surface with a permanent cautery spatula. Then, sterile scissors and tweezers were used to remove the upper external segment of the liver and to collect fragments of the internal tissue, avoiding contact with the external surface of the liver even if it was previously sanitized.

In addition, archive samples of chicken faeces (caecal contents) of broiler chickens from different flocks and farms in northeastern Spain collected from a slaughterhouse in 2015 and stored at −20°C were used to analyse the chicken faecal viromes.

### Homogenization of the liver samples

Ten grams of liver samples (requiring three livers from chickens of the same batch and farm) of Group 1 and 2 were homogenized in 40 ml of phage buffer. Tissues were disrupted using 1 and 3 mm glass beads (Hecht Assistant^®^, Sondheim vor der Rhön. Germany) following the manufacturer’s instructions and the homogenates were recovered.

At this point, the homogenates were used for the analysis of bacterial indicators, as described below. Subsequently, the homogenates were centrifuged at 4000 *g* for 25 min. The supernatant was collected and centrifugation was repeated under the same conditions. The final supernatant was filtered through 0.22 μm low protein‐binding polyethersulfone membranes (PES) (Millex ‐GP, Millipore, Bedford, MA). The filtrates were used for the analysis of somatic coliphages and the extraction of viral DNA.

### Culturable bacterial and viral indicators

Two groups of culturable indicators were analysed in the liver samples from groups 1 and 2. Total aerobic microorganisms and ampicillin (amp)‐resistant total aerobic microorganisms were evaluated in the homogenate of the livers for the presence of culturable bacteria. To do this, the homogenates were diluted 1/10 and 1/100. 0.1 ml of the homogenate or the dilutions were plated on Tryptone Soy agar (TSA) and TSA with amp (100 μg ml^−1^) and incubated at 37°C. Each homogenate was analysed in duplicate.

Somatic coliphages, proposed as viral indicators of faecal pollution (Jofre, 2007), were evaluated for the presence of infectious faecal viruses in the samples. To do this, 10 ml of homogenates were centrifuged for 15 min at 4000 *g* and the supernatants were filtered through 0.22 μm low protein‐binding polyethersulfone membranes (PES) (Millex ‐GP, Millipore, Bedford, MA). Decimal dilutions of the filtrates were performed and 1 ml of each was analysed by double agar layer in duplicate for the presence of somatic coliphages following the ISO standard method (Anonymous, 2000) that uses *E. coli* strain WG5 (ATCC 700078) as the bacterial host. Plates were incubated at 37°C for 18 h. Each homogenate was analysed in duplicate.

### Extraction of phage particles and removal of non‐packaged DNA

To extract the DNA of viral particles in the sample, regardless they were infective or not, the homogenates obtained as described above were treated with chloroform (1:10 (v/v)) to remove any extracellular vesicles. The samples were vortexed for 5 min and centrifuged at 16 000 *g* for 5 min. The supernatant was recovered and digested with DNase (100 units ml^−1^ of the supernatant) to eliminate any DNA that might be present in the samples outside the phage particles. DNase was inactivated by heating for 5 min at 75°C and an aliquot was taken as a control to verify the removal of non‐packaged DNA by qPCR amplification of bacterial *16S rRNA* and ARGs as described below. Additional controls were performed to confirm the correct inactivation of DNase by heat treatment, as described previously (Colomer‐Lluch *et al*., 2014).

Once the control was confirmed as negative, lysates were used to observe phage particles by electron microscopy and to extract the DNA in the phage particles (packaged DNA).

### Transmission electron microscopy

Viruses from the homogenates of liver samples of group 2 were purified by filtration, treated with chloroform as described above and 100× concentrated by protein concentrators (100‐kDa Amicon Ultra centrifugal filter units; Millipore, Bedford, MA). Seven microlitres of the concentrated phage suspension was dropped onto copper grids with carbon‐coated Formvar films and negatively stained with 2% ammonium molybdate (pH 6.8) for 1.5 min. Phages were visualized using a Jeol 1010 transmission electron microscope (JEOL Inc. Peabody, MA, USA) operating at 80 kV.

### Extraction of packaged DNA for ARG quantification

The protocol for DNA extraction was as previously described (Sambrook and Russell, 2001) with some modifications. After performing the chloroform and DNase treatments and obtaining negative results for ARGs and *16S rRNA* amplification, which ruled out the presence of non‐encapsidated DNA, capsids of the viral suspensions were degraded with proteinase K (20 mg ml^−1^) in 250 μl of proteinase K buffer and incubated for 1 h at 55°C. Packaged DNA was then extracted by the phenol–chloroform (1:1) (v:v) method and the aqueous phase was again treated with chloroform (1:1) (v:v), centrifuging at the same speed and time as in the previous step. The remaining phenol/chloroform was removed by placing the mixture in Phase Lock Gel Tubes (5‐Prime, Hucoa Erlöss, Madrid, Spain) for centrifugation following the manufacturer’s instructions. DNA was precipitated using 100% ethanol and 3 M sodium acetate and resuspended in a final volume of 100 µl of ultrapure water.

### qPCR analysis

The ARGs were evaluated by qPCR TaqMan assays (Table S1). The absence of bacterial *16S rRNA* was verified by qPCR using Power SYBR Green PCR Master Mix (Thermo Fisher Scientific, Waltham, MA, USA) (Table S1).

qPCR using TaqMan hydrolysis probes was conducted with the StepOne Real‐Time PCR System (Applied Biosystems, Waltham, MA, USA) in a 20 μl reaction mixture with the TaqMan^®^ Environmental Master Mix 2.0 (Applied Biosystems). The results were analysed with the Applied Biosystems StepOne™ Instrument program.

Six qPCR assays targeting ARGs were performed in 9 μl of the sample DNA or quantified DNA. The targets were clinically relevant genes that confer resistance to β‐lactam antibiotics (*bla*_TEM,_ *bla*_CTX‐M_‐_1_ group), a quinolone resistance gene (*qnrA*), *sul1*, which confers resistance to sulphonamides and is frequently found in environmental and clinical bacterial populations (Pruden *et al*., 2012), *armA*, which encodes aminoglycoside resistance and is widely distributed in *Enterobacteriaceae* (Galimand *et al*., 2005), and *tetW,* conferring resistance to tetracycline, an antibiotic commonly used in intensive poultry farming (Mehdi *et al*., 2018).

For quantification, serial dilutions of gBlocks™ Gene Fragments (Integrated DNA Technologies, Coralville, USA) of known concentration were used to generate the standard curves. All samples were run in triplicate (including the standards and negative controls). The number of gene copies (GC) was defined as the mean of the triplicate data obtained. To quantify the ARGs, we considered the results obtained within the threshold cycle (Ct) and within the limit of quantification (LOQ). This was determined by the last valid Ct for each ARG assay (GC μl^−1^ are shown in Table S1) in the standard curve that was consistent in the diverse replicates. gBlocks were also used as positive controls.

### Metagenomic analysis

Ten liver samples from group 2 (10 g) were pooled and used for sequencing the viral fraction (Virome PL). Chicken faecal samples were divided into two pools (chicken faeces HP1 and HP2). For each pool of liver or faeces, 10 g was homogenized together in 40 ml of phage buffer using a horizontal agitator at 800 rpm for 10 min. Viruses were recovered from the samples after filtration, chloroform and DNAse treatment as described above. Before breaking the capsids, the removal of non‐packaged DNA was confirmed by qPCR amplification of bacterial *16S rRNA* as a control.

The use of phenol in the DNA extraction protocol described above meant that the DNA quality was insufficient for metagenomic analysis, as previously reported (Fernández‐Orth *et al*., 2019). Therefore, the phage lysates were used to extract the DNA for metagenomic analysis using the QIAmp^®^ Viral RNA Mini Kit (Hilden, Germany), which broke down the capsids and allowed the viral DNA to be recovered in a final volume of 80 μL of bidistilled water. The DNA from the samples was pooled and further purified using the DNA Clean & ConcentratorTM‐5 Kit (Zymo Research, Irving, CA, USA) to a final volume of 50 μl, which contained the required DNA concentration for subsequent sequencing analysis.

### Sequencing

The DNA concentration for metagenomics was evaluated using a Qubit^®^ Fluorometer (Life Technologies, CA, USA) and the DNA quality was further confirmed using the 2100 Bioanalyzer system (Agilent Technologies, CA, USA). 0.2 ng μl^−1^ of DNA was used to prepare the libraries. The DNA was fragmented and used to prepare the libraries with the Nextera XT Kit (Illumina. Inc. San Diego, CA, USA) protocol for paired‐end libraries (2 × 150 bp). For extension, 14 PCR cycles of 2.5 min were performed to increase the tagmentation process. Libraries were purified using AmPure beads (Beckman Coulter Inc., California, USA), checked for the distribution and size of the fragments in a 2100 Bioanalyzer and the Agilent High Sensitivity DNA Chip (DNA 1000) (Agilent Technologies, CA, USA) and quantified in a Quantus™ Fluorometer (Promega, WI, USA). An equimolar pool of the seven samples was sequenced in a NextSeq Illumina in a High Output run of 300 cycles.

### Bioinformatic analysis

Reads were analysed with FastQC (version 0.11.9) and summarized with MultiQC (version 1.11). As the files showed low sequence quality, high duplication levels, overrepresented sequences and the presence of adapters, a preprocessing step was performed. First, the duplications for each file were removed with Seqkit (v0.16.1). Then, the adapters for the paired‐end files were cut with Trimmomatic (v0.39). Finally, the remaining duplicates and the sequences with quality below 20 for the paired‐end files were removed with PRINSEQ (v0.20.4).

Reads were *de novo* assembled with SPAdes (version 3.15.3), specifically MetaSPAdes (Nurk *et al*., 2017), which is recommended when assembling metagenomics data sets. The obtained scaffolds from this assembly were used in the following analysis.

Contigs were taxonomically classified with Kraken2 (version 2.0.7 beta) (Wood and Salzberg, 2014).

The presence of ARGs in the viromes was determined by the specific software ResFinder (version 4.1) (Zankari *et al*., 2012). Since this software is very restrictive, ARGs search was completed with CARD database (McArthur *et al*., 2013) and Prokka (version 1.14.5) (Seemann, 2014) with the terms ‘resist’, ‘betalactam’, ‘penicillin’ which includes antibiotic as well as other resistances.

Comparison of sequences between the chicken liver and faecal viromes was also done by BLAST+ (version 2.10.1). For this, two Blastn queries (PL vs. HP1 and PL vs. HP2) were performed. The PL sequences were inputted as a query, and the HP1 and HP2 files were the database where the query was searched in each comparison. It was determined that the output matches must have an E‐value < 1^‐10^, which indicated an almost identical match.

### Data availability

The metagenomic data set generated was deposited in BioProject (PRJNA782068).

### Statistics

Statistical tests were performed using the Statistical Package for Social Science Software (SPSS). A paired Student’s‐*t* test (*P* < 0.05) was used to evaluate the differences between ARGs in sterile and non‐sterile liver samples.

## Conflict of interest

None declared.

## Supporting information


**Table S1**. Oligonucleotides used in this study.
**Table S2**. Percentatges of viral species in fecal viromes.

**Table S3**. Common species detected in the two viromes compared (PL vs HP1 or PL vs HP2).
