# Supplementary material for: Chicken liver is a potential reservoir of bacteriophages and phage‐derived particles containing antibiotic resistance genes
Source: Microb Biotechnol. 2022 Apr 29;15(9):2464–75. doi: 10.1111/1751-7915.14056 (PMC9437878; doi:10.1111/1751-7915.14056)
Supplement: Supplementary file 1 — Table S1. Oligonucleotides used in this study. Table S2. Percentatges of viral species in fecal viromes. [file MBT2-15-2464-s002.docx]

**Supplementary material**

**Table S1**-Oligonucleotides used in this study

| **Target gene** | **Reaction** | **Oligonucleotide** | **Sequence** | **Amplimer (bp)** | **LOQ (GC)** | **Reference** |
| --- | --- | --- | --- | --- | --- | --- |
| *bla*_TEM_ | qPCR | UP | CACTATTCTCAGAATGACTTGGT | 85 | 7.6 | [1] |
|  |  | LP | TGCATAATTCTCTTACTGTCATG |  |  |  |
|  |  | TaqMan TEM | FAM-CCAGTCACAGAAAAGCATCTTACGG-MGBNFQ |  |  |  |
| *bla*_CTX-M-1_ | qPCR | UP | ACCAACGATATCGCGGTGAT | 101 | 8.4 | [2] |
|  |  | LP | ACATCGCGACGGCTTTCT |  |  |  |
|  |  | TaqMan CTX-M-1 | FAM–TCGTGCGCCGCTG-MGBNFQ |  |  |  |
| *sul1* | qPCR | UP | CCGTTGGCCTTCCTGTAAAG | 67 | 5.9 | [3] |
|  |  | LP | TTGCCGATCGCGTGAAGT |  |  |  |
|  |  | TaqMan sul1 | FAM-CGAGCCTTGCGGCGG-MGBNFQ | 62 | 1.9 | [4] |
| *tetW* | qPCR | UP | GACGGACACCATGTTTTTGGA |  |  |  |
|  |  | LP | AGGAAGTGACTGCCGCTTGA |  |  |  |
|  |  | TaqMan tetW | FAM-AGCGTGGGATTACCA-MGBNFQ |  |  |  |
| *armA* | qPCR | UP | GAAAGAGTCGCAACATTAAATGACTT | 94 | 33.4 | [5] |
|  |  | LP | GATTGAAGCCACAACCAAAATCT |  |  |  |
|  |  | TaqMan armA | FAM-TCAAACATGTCTCATCTATT-MGBNFQ |  |  |  |
| 16SrDNA | qPCR | 338F | ACTCCTACGGGAGGCAGCAG | 236 |  | [6] |
|  |  | 518R | ATTACCGCGGCTGCTGG |  |  |  |

**Table S2.-**Percentatges of viral species in fecal viromes

|  | **HP1** | **HP2** |
| --- | --- | --- |
| *Myoviridae* | 41 | 27 |
| *Siphoviridae* | 22 | 23 |
| *Podoviridae* | 10 | 12 |
| *Drexleviridae* | 10 | 10 |
| *Herelleviridae* | 4 | 4 |
| *Autrographviridae* | 4 | 9 |
| Other Caudovirales | 2 | 3 |
| Aviadenovirus | 4 | 8 |
| Other viruses | 3 | 4 |

**Supplementary references**

1. Lachmayr, K.L.; Kerkhof, L.J.; Dirienzo, A.G.; Cavanaugh, C.M.; Ford, T.E. Quantifying nonspecific TEM beta-lactamase (blaTEM) genes in a wastewater stream. *Appl. Environ. Microbiol.* **2009**, *75*, 203–11, doi:10.1128/AEM.01254-08.

2. Colomer-Lluch, M.; Jofre, J.; Muniesa, M. Antibiotic resistance genes in the bacteriophage DNA fraction of environmental samples. *PLoS One* **2011**, *6*, e17549.

3. Calero-Cáceres, W.; Melgarejo, A.; Colomer-Lluch, M.; Stoll, C.; Lucena, F.; Jofre, J.; Muniesa, M. Sludge as a potential important source of antibiotic resistance genes in both the bacterial and bacteriophage fractions. *Environ. Sci. Technol.* **2014**, *48*, 7602–11, doi:10.1021/es501851s.

4. Blanco-Picazo, P.; Roscales, G.; Toribio-Avedillo, D.; Gómez-Gómez, C.; Avila, C.; Ballesté, E.; Muniesa, M.; Rodríguez-Rubio, L. Antibiotic Resistance Genes in Phage Particles from Antarctic and Mediterranean Seawater Ecosystems. *Microorganisms* **2020**, *8*, 1293, doi:10.3390/microorganisms8091293.

5. Quirós, P.; Colomer-Lluch, M.; Martínez-Castillo, A.; Miró, E.; Argente, M.; Jofre, J.; Navarro, F.; Muniesa, M. Antibiotic resistance genes in the bacteriophage DNA fraction of human fecal samples. *Antimicrob. Agents Chemother.* **2014**, *58*, 606–9, doi:10.1128/AAC.01684-13.

6. Weisburg, W.G.; Barns, S.M.; Pelletier, D.A.; Lane, D.J. 16S ribosomal DNA amplification for phylogenetic study. *J. Bacteriol.* **1991**, *173*, 697–703.

The metagenomic data set generated was deposited to BioProject (PRJNA782068). Data can be checked by the reviewers under the following link:

<https://dataview.ncbi.nlm.nih.gov/object/PRJNA782068?reviewer=mnenvujmgm8d9jfqjvjoud28ut>

The URL will expire when this BioProject is publicly-released.
